# New York College of Veterinary Surgeons

**Published:** 1899-05

**Authors:** 


### COMMENCEMENT EXERCISES.

### NEW YORK COLLEGE OF VETERINARY SURGEONS.

No formal graduating exercises were held at the college. On April 1st the following students were presented with diplomas: William Malcolm MacKellar, 167 Clymer Street, Brooklyn, N. Y.; Francis C. Edmonds, Glen Cove, N. Y.; Robert H. Twitty, 329 Clinton Street, Buffalo, N. Y.; Stephen J. Hanlon, 65 Clark Street, Brooklyn, N. Y.; George B. Morse, 154 East Fifty-seventh Street, N. Y.; Alphonso J. Doncourt, Fourth Avenue, Williamsbridge, N. Y.; William P. Grimes and Wallace M. Gill, of New York City.

William Malcolm MacKellar was awarded the gold medal for the best senior examination and the pocket-case of instruments for the best practical examination. Mr. Daniel J. Mangan, a second-year student, received a hypodermic syringe for the best examination in materia medica, and Charles Joseph Jones, a first-year student, received the silver medal for the best junior examination.

